# Bulk and Single‐Cell Transcriptomic Reveals Shared Key Genes and Patterns of Immune Dysregulation in Both Intestinal Inflammatory Disease and Sepsis

**DOI:** 10.1111/jcmm.70415

**Published:** 2025-02-24

**Authors:** Chao Liu, Jinliang Liu, Yitian Yang

**Affiliations:** ^1^ Department of Infectious Diseases, The Second Affiliated Hospital Zhejiang University School of Medicine Zhejiang Hangzhou China; ^2^ Department of Anesthesiology and Perioperative Medicine, Henan Provincial People's Hospital People's Hospital of Zhengzhou University Zhengzhou Henan China

**Keywords:** CD14^+^ monocytes, differentially expressed genes, IBD, IL1B^+^ macrophages, sepsis, single cell transcriptome

## Abstract

Inflammatory bowel disease (IBD) and Sepsis are both characterised by immune dysregulation. Notably, IBD is a factor in the increase in septic infections. However, these two conditions' shared molecular and pathophysiological mechanisms remain unclear. We used ‘limma’ and ‘WGCNA’ analyses to identify common DEGs between these two conditions. Single‐cell RNA sequencing further assessed immune cell heterogeneity. We used machine learning algorithms to construct and identify diagnostic markers for Sepsis, which we then validated using receiver operating characteristic curve (ROC) analysis. A mouse model of IBD combined with Sepsis was constructed, and real‐time PCR and western blot validated the expression of BCL2A1 and CEBPB. It was found that 58 shared DEGs identified in both IBD and Sepsis were highly enriched in immune and inflammation‐related pathways. Single‐cell analysis revealed that CD14^+^ monocytes (or IL1B^+^ macrophages) primarily express these hub genes. Both conditions significantly increased the proportion of this cell type compared to healthy controls. Finally, BCL2A1 and CEBPB were identified as potential biomarkers that have strong diagnostic potential. Furthermore, we confirmed that levels of BCL2A1 and CEBPB were elevated in mice with IBD complicated by Sepsis through real‐time PCR and observed that IBD exacerbates the progression of Sepsis. We conclude that IL1B^+^ macrophages expressing high levels of these hub genes play a key role in the immune dysregulation associated with both IBD and Sepsis. The overlapping gene expression and pathway alterations in these cells indicate shared common molecular mechanisms, suggesting new strategies for targeted therapeutic interventions.

## 1 Introduction

Sepsis is a life‐threatening organ dysfunction arising from aberrant immune responses to infection and is characterised by high incidence, complex pathogenesis, severe illness and high mortality [1, 2]. According to a report in 2017, approximately 50 million people were diagnosed with Sepsis globally, resulting in over 11 million deaths, accounting for one fifth of global mortality cases [3]. The aetiology of this disease is multifactorial, involving genetic predisposition, environmental triggers and a range of immune dysfunctions. Although clinical interventions such as antibiotics, intravenous fluids and comprehensive organ support have enabled an increasing number of patients to survive sepsis treatment, the mortality rate from Sepsis remains alarmingly high [4]. Therefore, it is essential to explore the biological mechanisms associated with Sepsis and to identify potential biomarkers.

The aetiology of Sepsis is multifactorial, involving genetic predisposition, environmental triggers and a range of immune dysfunctions [5, 6]. A retrospective study revealed that individuals with inflammatory bowel disease (IBD) are at increased risk of serious infections [7]. IBD is a chronic, recurrent autoimmune‐related inflammatory disease involving the ileum, rectum and colon, including ulcerative colitis (UC) and Crohn's disease (CD). The incidence of IBD has been increasing in recent years, with a prevalence rate ranging from 156 to 291 cases per 100,000 people, which constitutes a global burden [8]. The causes of inflammatory bowel disease (IBD) are complex and may involve chronic systemic inflammation, with factors such as tumour necrosis factor‐α (TNF‐α) and interleukins (ILs) potentially contributing to the destruction of the intestinal epithelium [9, 10]. Furthermore, the activated immune response mediated by inflammatory cytokines can be an important risk factor for Sepsis [11, 12]. Besides, scholars have also found that histologic inflammation associated with IBD is an independent risk factor for serious infections, including sepsis [7]. While both IBD and Sepsis are characterised by immune dysfunction, the shared mechanisms underlying these conditions have not yet been fully elucidated. Identifying common genetic and cellular alterations may lead to significant insights that facilitate the development of more effective therapeutic strategies and ultimately enhance patient outcomes.

In this study, we used bulk and single‐cell transcriptome data to look for molecular and cellular connections between IBD and Sepsis‐related gene expression. Our research explores the underlying immune mechanisms in cases of Sepsis complicated by IBD. Furthermore, we developed a screening model based on biomarkers, which may improve earlier diagnosis of patients with comorbidities and provide a new strategy for treatment.

## 2 Materials and Methods

### 2.1 Data Collection and Processing

**FIGURE 1:**
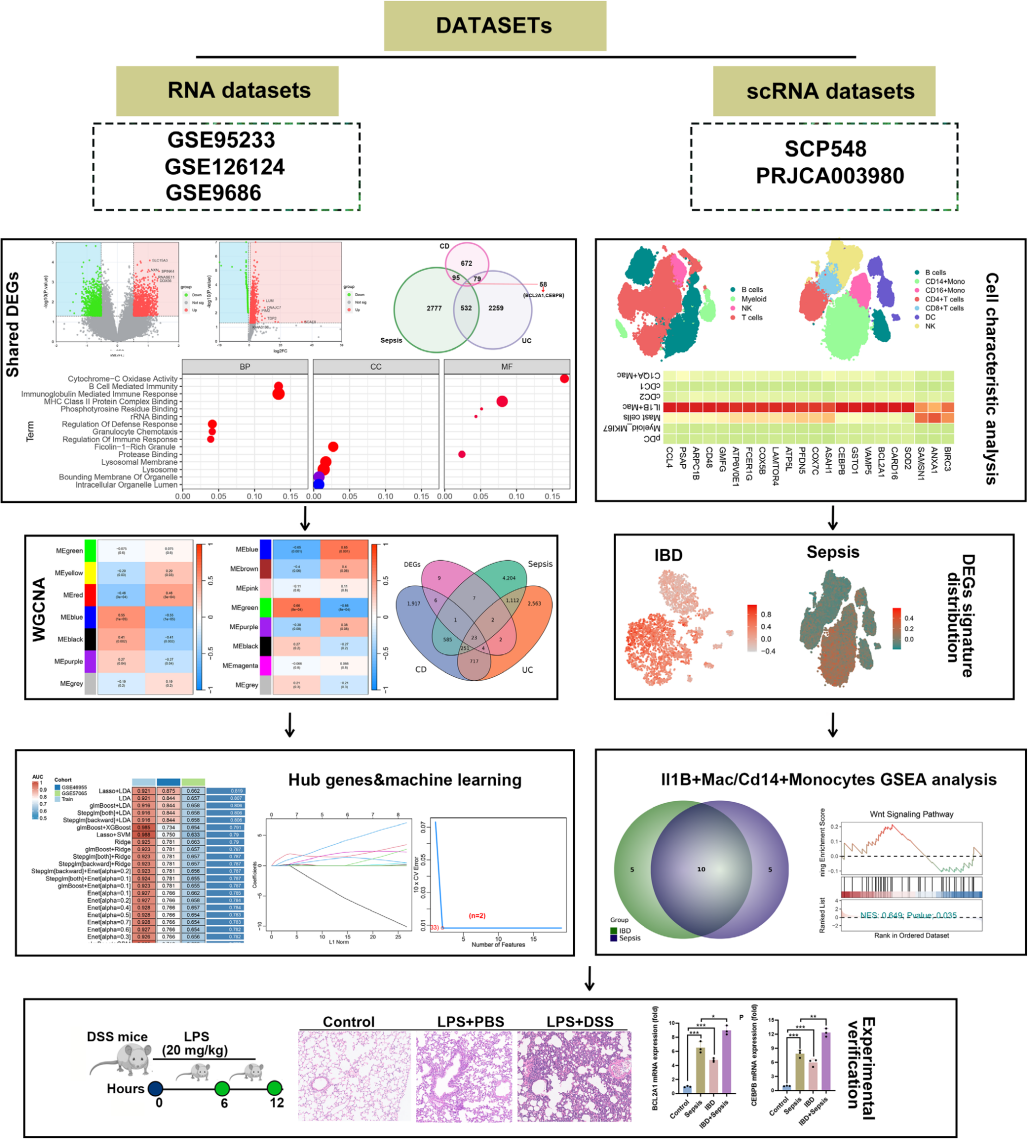
The schematic diagram of this work.

The Bulk RNA datasets for Sepsis (GSE95233 [13]) and IBD (GSE126124 [14], GSE9686 [15] and GSE36807 [16] were downloaded from the GEO database (https://www.ncbi.nlm.nih.gov/geo/). GSE126124, using the GPL6244, including 37 Crohn's disease (CD) patients and 21 healthy controls. GSE9686 and GSE36807, using GPL5760 and GPL570, respectively, included 20 UC patients and 15 healthy controls. For the sepsis cohort, GSE95233, using the GPL6244, including 102 sepsis patients and 21 healthy controls. Additionally, we also analysed the single‐cell RNA sequencing dataset SCP548, consisting of 29 sepsis samples and 16 healthy samples (https://singlecell.broadinstitute.org/single_cell/study/SCP548/), as well as BioProject: PRJCA003980 from the GSA database [17], specifically targeting CD45^+^ mucosal immune cells, including 12 individuals with IBD (6 CD and 6 UC) and three healthy controls. Additionally, two other expression profile data and clinicopathological information for individuals with Sepsis and IBD (GSE46955 [18], GSE57065 [19]) and GSE75214 [20]) were collected from the GEO. The schematic diagram of the whole workflow in this study is presented in Figure 1.

### 2.2 Identification of the Common DEGs Between IBD and Sepsis

The ‘limma’ package (version 3.46.0) [21] in R (version 4.0) was performed to identify differentially expressed genes (DEGs) for the datasets GSE9523. Genes with |log2 Fold change| > 0.585 and adjusted *p* < 0.05 were identified as differentially expressed genes (DEGs). When using ‘limma’ to identify DEGs, we assume that gene expression levels follow a normal distribution and that the samples are independent. We thus performed batch correction and normalised the gene expression data to ensure that these assumptions are satisfied as much as possible, thereby enhancing the stability and reliability of the results. In the analysis of UC datasets, the merge function in R was employed to integrate the GSE9686 and GSE36807 datasets. Subsequently, data normalisation was conducted, and differentially expressed genes (DEGs) were identified using the ‘limma’ package, with criteria set at |log2FC| > 0.585 and *p*‐value < 0.05 [22, 23, 24]—Furthermore, The online tool Jven was employed to obtain the shared DEGs between IBD and Sepsis.

### 2.3 Gene Ontology (GO), Disease Ontology, and Pathway Analysis

To further investigate the potential biological functions of these common DEGs and their associations with diseases, the ‘clusterProfiler’ package (version 4.14.4) [25] was employed to conduct GO and disease ontology (DO) analyses, along with enrichment analysis of the shared DEGs, using a threshold *p* < 0.05. Additionally, the Enrichr database (https://maayanlab.cloud/Enrichr/) was utilised to identify significant signalling pathways associated with these shared differentially expressed genes (DEGs), including the Kyoto Encyclopedia of Genes and Genomes (KEGG) pathways, WikiPathways, Reactome and Hallmark. A cut‐off of *p* < 0.05 was applied.

### 2.4 WGCNA Analysis

Similar to previously published studies [26], when conducting weighted gene co‐expression network analysis (WGCNA), we assume that the relationships between genes are linear and that the correlations among samples can capture the potential associations between genes and phenotypes. Therefore, WGCNA was utilised to analyse the IBD (GSE126124, GSE9686 and GSE36807) and Sepsis (GSE95233) datasets employing the ‘WGCNA’ package (version 1.16.0) in R software to identify gene modules correlated with inflammatory bowel disease (IBD) and Sepsis. Subsequently, a co‐expression network was constructed by applying a soft thresholding approach to ensure a high scale‐free topology fitting index (R^2^) and optimal mean connectivity among the genes. The minimum number of gene modules for the dynamic tree cut and module identification process was set to 100. Clinical characteristic data for Sepsis and IBD samples were extracted from the IBD and Sepsis datasets, and the relationships between gene modules and clinical traits were visualised through a heatmap.

### 2.5 Construction of Diagnostic Model

A total of 10 diverse machine learning algorithms were integrated, including least absolute shrinkage and selection operator (LASSO), Gradient Boosting Machine (GBM), random forest (RSF), partial least squares regression for Cox (plsRcox), stepwise Cox (StepCox), supervised principal components (SuperPC), ridge, survival support vector machine (Survival‐SVM), CoxBoost and elastic network (Enet). Among these, the first‐step dimensionality reduction and variable screening were carried out using RSF, LASSO, CoxBoost, StepCox (both directions) and StepCox (backward direction). These methods were then combined with other algorithms to produce a total of 101 machine learning algorithm combination [27, 28]. We adopted a sequential approach that comprised the identification of prognostic variables through univariate Cox regression modelling, the development of predictive models based on the GSE95233 cohort and the subsequent validation of these models using other external and independent datasets (GSE57065 and GSE46955). The Harrell's Consistency Index (C‐index) was calculated for model selection. The model with the highest average C‐index in all cohorts was identified as the optimal model.

### 2.6 ROC Curve

To further evaluate the diagnostic model for Sepsis, Receiver Operating Characteristic (ROC) curve analyses were performed on gene expression data from the GSE95233, GSE57065 and GSE46955 datasets, utilising the pROC package (Robin et al., 2011). Moreover, we specifically used ROC curve analyses on the GSE95233, GSE57065, GSE46955, GSE126124, GSE9686, GSE36807 and GSE75214 datasets to assess the diagnostic value of hub genes in Sepsis and IBD. We performed the Area Under the Curve (AUC) to compare the discriminative abilities of the LASSO models in diagnosing Sepsis with the diagnostic performance of individual genes associated with Sepsis and IBD.

### 2.7 Analysis of Single‐Cell RNA Sequence Data

The Seurat software package (version 4.3.0, https://satijalab.org/seurat/) was employed to normalise the expression matrix and obtain scaled data by considering the UMI counts of each sample and the percentage of mitochondria genes. Subsequently, the top 2000 highly variable genes (HVGs) were selected for sample integration using the ‘FindVariableGenes’ function, and the ‘RunPCA’ function was used to analyse the principal component of these HVGs [29]. The ‘Harmony’ package was then employed to eliminate batch effects across different samples [30]. Then, we set the resolution to 1.0 for the purpose of identifying cell types within all cell populations, and the collected cells were then projected into a two‐dimensional space via the ‘RunTSNE’ function. The ‘Dimplot’ function was employed to visualise the cell clusters [17]. The ‘UCell’ package was then employed to calculate the enrichment scores of the hub genes across all cells [31]. Differential expression analysis was performed utilising the ‘FindMarkers’ function in Seurat to identify differentially expressed genes (DEGs) across distinct disease states. The default thresholds of the ‘FindMarkers’ function for DEG identification were established as follows: |average fold change| ≥ 0. 25 and *p* < 0.05 [32].

### 2.8 Functional Enrichment Analysis

To elucidate the functional role of comorbid macrophages or monocytes identified from patients with Sepsis and IBD compared to healthy controls, we performed Gene Set Enrichment Analysis (GSEA) and KEGG enrichment analysis. First, the DEGs identified in macrophages or monocytes derived from patients with Sepsis and IBD were compared to healthy controls using the ‘FindMarkers’ function [32]. Subsequently, the KEGG pathways with significant enrichment were ranked and visualised based on the Net Enrichment Score (NES), gene counts and associated *p*‐values using the ‘clusterProfiler’ and ‘ggplot2’ packages.

### 2.9 Mice Experiment

Female C57BL/6J mice (6–8 weeks old) were purchased from Shanghai SLAC Laboratory Animal Co. Ltd. (Shanghai, China) and housed at Zhejiang University's Laboratory Animal Center (Hangzhou, China). The mice were maintained in a specific pathogen‐free facility, and the experimental protocols were approved by the Animal Care and Use Committee of the Zhejiang University of Medicine (Hangzhou, China). Female mice at 8–10 weeks were used for experiments. Wild‐type (WT) mice were randomly assigned to two groups: control (Ctrl) and DSS. The control group received distilled water for 32 days, while the DSS group was given drinking water with 3% dextran sodium sulfate (DSS; molecular weight 30,000–50,000 g/mol; MP Biomedicals, Canada), refreshed every 2 days. After 1 week, DSS concentration was adjusted daily to 0%–1.5% to induce mild to moderate inflammation, as assessed by the disease activity index (DAI), which included blood presence in stool, weight loss and faecal characteristics. This regimen was sustained until the end of the experiment to model chronic intestinal Inflammation from day 8 to day 32. After 32 days of treatment, these mice received an intraperitoneal injection of either phosphate‐buffered saline (PBS) or LPS (20 mg/kg, Solarbio) [33]. Twelve hours after LPS treatment, the mice were killed, and peripheral blood was collected into centrifuge tubes. Peripheral blood mononuclear cells (PBMCs) were isolated using a mouse Peripheral Blood Lymphocyte Separation Kit (Beyotime) following the manufacturer's instructions.

## 3 Real‐Time PCR

Total RNA was isolated using TRIzol reagent (Thermo Fisher Scientific) following the manufacturer's instructions. The cDNA synthesis was conducted in accordance with the protocols provided by a kit from cwbio (China). The endogenous reference for mRNA quantification was GAPDH. SYBR Green (Tsingke) was employed to conduct real‐time PCR assays on an Applied Biosystems 7500 system (Thermo Fisher Scientific). The specific primers used for these assays are detailed in Table S1.

### 3.1 Transfection of ASOs

Cholesterol‐conjugated‐BCL2A ASOs and CEBPB ASOs or their corresponding controls (GenePharma, Shanghai, China) using the TransIT‐TKO Transfection Reagent (Mirus Bio, Madison, WI, USA) according to the manufacturer's instructions.

### 3.2 Statistical Analysis

To evaluate the differences between the groups, an unpaired Student's t‐test was implemented for comparisons between two groups, while a one‐way or two‐way ANOVA was implemented for comparisons among multiple groups. All data are expressed as the mean ± SD values and were analysed using GraphPad Prism 8.0 (GraphPad Software Inc., San Diego, CA, USA). A *p* < 0.05 represented statistical significance. The correlations in our data were assessed using Pearson's correlation analysis.

## 4 Results

### 4.1 Identification of Common DEGs Between Sepsis and IBD

To explore the possible relationship between people with IBD and Sepsis, we analysed four RNA‐seq datasets (GSE126124, GSE9686, GSE36807 and GSE95233) to identify differentially expressed genes (DEGs) utilising an absolute log2 fold change (log2FC) threshold of 0.585 and an adjusted *p*‐value criterion of 0.05. In the sepsis cohort, 3367 DEGs were obtained, including 1898 upregulated and 1469 downregulated. For IBD, a total of 904 differentially expressed genes (DEGs) were identified, including 438 upregulated and 466 downregulated genes, from the CD cohort, while 2849 DEGs were identified from the UC cohort, consisting of 2088 upregulated and 761 downregulated genes, utilising the ‘limma’ package (Figure S1A–C). After that, a Venn diagram and heatmap were used to identify a total of 58 shared DEGs in patients with Sepsis and IBD (Figure S1D,E). Therefore, these two diseases may be interconnected regarding their onset and course as their shared DEG. Subsequently, these shred DEGs were selected for further investigation.

### 4.2 GO, DO, And Pathway Enrichment Analysis of the Shared DEGs

To reveal the potential biological processes, molecular functions and related diseases associated with these genes, GO and DO enrichment analyses were performed on the shared DEGs using the ‘cluster profile’ package. The five Gene Ontology categories that are most substantially enriched are displayed in a bubble plot and arranged based on the *p*‐value. The results revealed that these 58 shared DEGs were significantly involved in the regulation of immune response, B cell or immunoglobulin immunity, MHC Class II protein complex binding, granulocyte chemotaxis, lysosomal membrane and protease or rRNA binding (Figure S1F and Table S2). Besides, the DO analysis results indicated that prevalent DEGs were markedly concentrated in autoimmune illnesses, including Inflammation disease, Sepsis, Inflammatory Bowel Diseases and Bacterial Infections (Figure S2C and Table S3). Furthermore, we investigated the pathways enriched by these shared DEGs in four databases and showed that these common DEGs were found to be involved in various pathways, including the NF‐κB, TNF, Toll‐like receptor, NOD‐like receptor, Th17 cell differentiation, Inflammatory Response, IL‐2/STAT5 Signalling, Immune System and Cytokine Signalling (Figure S2A,B and Tables S4 and S5). The data indicate that these shared DEGs were primarily involved in functional enrichment associated with inflammation and immune responses.

### 4.3 Identification of Modules With Highest Positive Correlation Disease States

**FIGURE 2:**
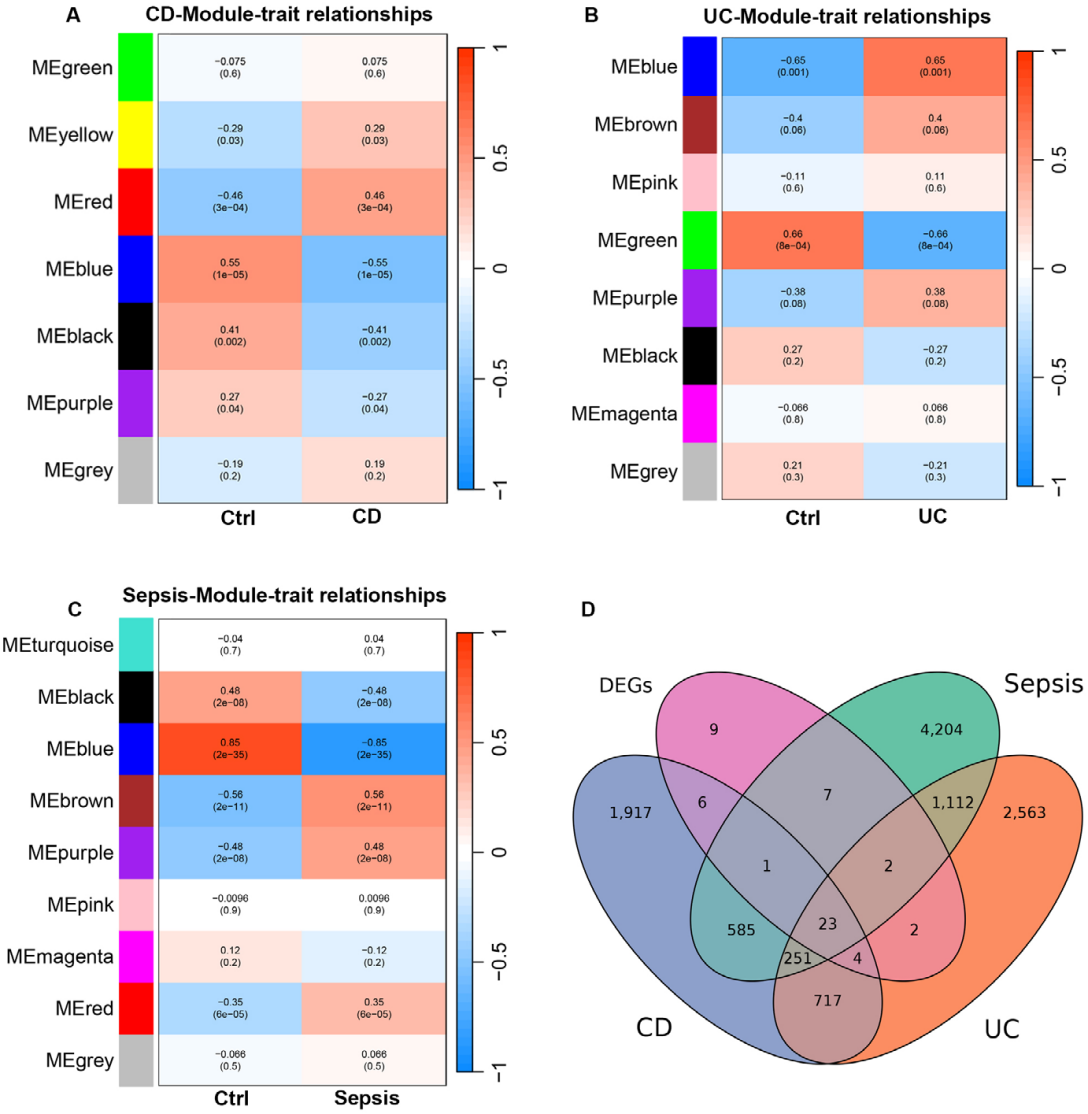
Identification of modules associated with clinical traits of IBD and Sepsis via WGCNA. (A–C) The heatmap illustrates the relationships between modules and traits in CD (A), UC (B), and Sepsis (C). In this heatmap, each row represents a module, while each column signifies a clinical trait. Each cell displays the corresponding correlation and *p*‐value between the modules and the different clinical traits. (D) The Venn diagrams illustrate the hub genes that are co‐expressed in shared DEGs and modules with the highest positive correlation in IBD and sepsis datasets.

We then conducted a WGCNA analysis to identify hub genes that exhibited the most significant correlation with disease conditions (including Sepsis and IBD). The soft threshold power for the scale‐free network was established at 11, 10 and 6, respectively. (Figure S3A,C,E). Based on threshold power and average linkage hierarchical clustering, we identified 7, 8 and 9 modules identified in CD, UC and Sepsis, respectively (Figure 2A–C and Figure S3B,D,F). Pearson correlation coefficient was used to analyse each module and sample characterisation; the yellow and red modules were significantly positively correlated with CD (r = 0.29 and 0.46, *p* < 0.05). For UC patients, the blue modules were significantly positively correlated with CD (*r* = 0.65, *p* < 0.05). For Sepsis patients, the brown, purple and red modules were closely positively correlated with sepsis patients (*r* = 0.56, 0.48 and 0.35, *p* < 0.05). After that, a total of 23 common DEGs, identified as hub genes due to their strong positive correlation with both Sepsis and IBD, were identified (Figure 2D).

### 4.4 Hub Gene Expression Was Elevated in IL1B ^+^ Macrophages From IBD Patients

**FIGURE 3:**
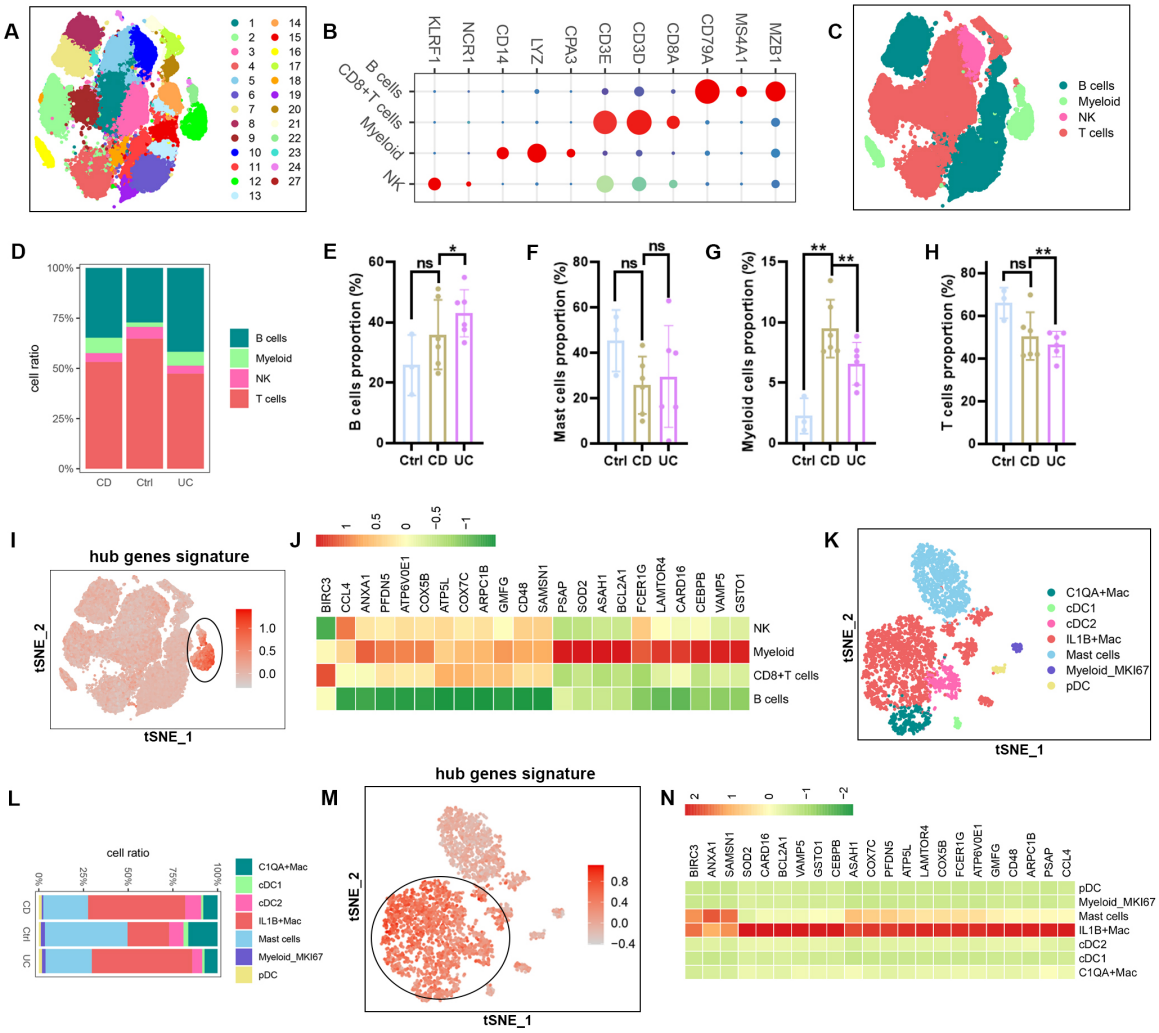
Immunological characterisation of DEGs in the single cell atlas of IBD patients. (A) tSNE visualisation of the clusters of immune cells. (B) The dot plot shows the annotation markers for the cell clusters. (C) tSNE plots showing the main cell types of IBD samples. (D)The bar chart depicts the relative frequency of each cell type across different groups. (E–H) Comparison of cell type proportions in different groups. (I) The plot of tSNE showing the module score distribution of DEGs modules in different cell types. (J) Heatmap visualisation of the expression patterns of the shared DEGs in each cell type. (K) tSNE map showing the main macrophage subgroups in IBD. (L)The bar chart visualisation of the relative frequency for each subpopulation of macrophage across different groups. (M) Module score distribution in tSNE space for these DEGs modules was evaluated using ‘UCell’ in macrophage subgroups. (N) Heatmap visualisation of the expression patterns of the common DEGs in macrophage subgroups. *p < 0.05; **p < 0.01; ns, not significant.

To deeply explore these 23 shared hub genes' expression profiles across different cell types in IBD, we performed scRNA‐seq analysis. A total of 27 clustering units were identified in the cells utilising graph‐based clustering approaches and uniform manifold approximation and projection (UMAP) for dimensionality reduction (Figure 3A). Subsequently, four major cell types were identified in these cell populations based on known markers. Clusters 7, 8, 14, 13, 6, 11 and 19 are termed as B cells. Clusters 12, 22 and 16 were defined as myeloid cells, clusters 1, 5, 3, 21, 17, 20, 4, 18, 2 and 9 were termed as T cells, and cluster 10 was defined as NK cells (Figure 3B,C). We observed a significant increase in the proportion of myeloid cells among these cell types in IBD patients compared to healthy controls (Figure 3D–H). Next, we used the ‘UCell’ package to evaluate module scores based on 23 hub genes, aiming to thoroughly assess the distribution of these shared hub genes across these cells. In IBD patients, myeloid cells had the highest module scores, corroborating the heatmap data that highlighted the elevated expression of these hub genes in myeloid cells compared to other cells (Figure 3I,J). To further elucidate the role of these 23 hub genes in the myeloid cells of IBD patients, we re‐clustered the myeloid cells and then identified 17 clusters (Figure S4A). A total of seven main cell types were obtained based on the classic cell markers in these clusters of myeloid cells (Figure S4B,C and Figure 3K). We observed a significant increase in the proportion of IL1B^+^macrophages in IBD patients compared to healthy controls, while C1QA^+^ macrophages were fewer compared with healthy controls (Figure 3L and Figure S4D–J). We assessed the module scores of these genes across several cell types using the ‘UCell’ package. tSNE visualisation revealed that IL1B^+^ macrophages had the highest module scores among these cell types, corroborating the heatmap data indicating the elevated expression of several hub genes in IL1B^+^ macrophages (Figure 3M,N). Besides, the heatmap of gene expression indicated that these hub genes were significantly upregulated in inflammatory IL1B^+^ macrophages from patients with IBD compared to control samples (Figure S4K). Consequently, these data indicate that these hub genes are active in IL1B^+^ macrophages and may significantly contribute to the pro‐inflammatory response in IBD.

### 4.5 Accumulation of the Hub Genes in CD14 ^+^ Monocytes in Sepsis Patients

**FIGURE 4:**
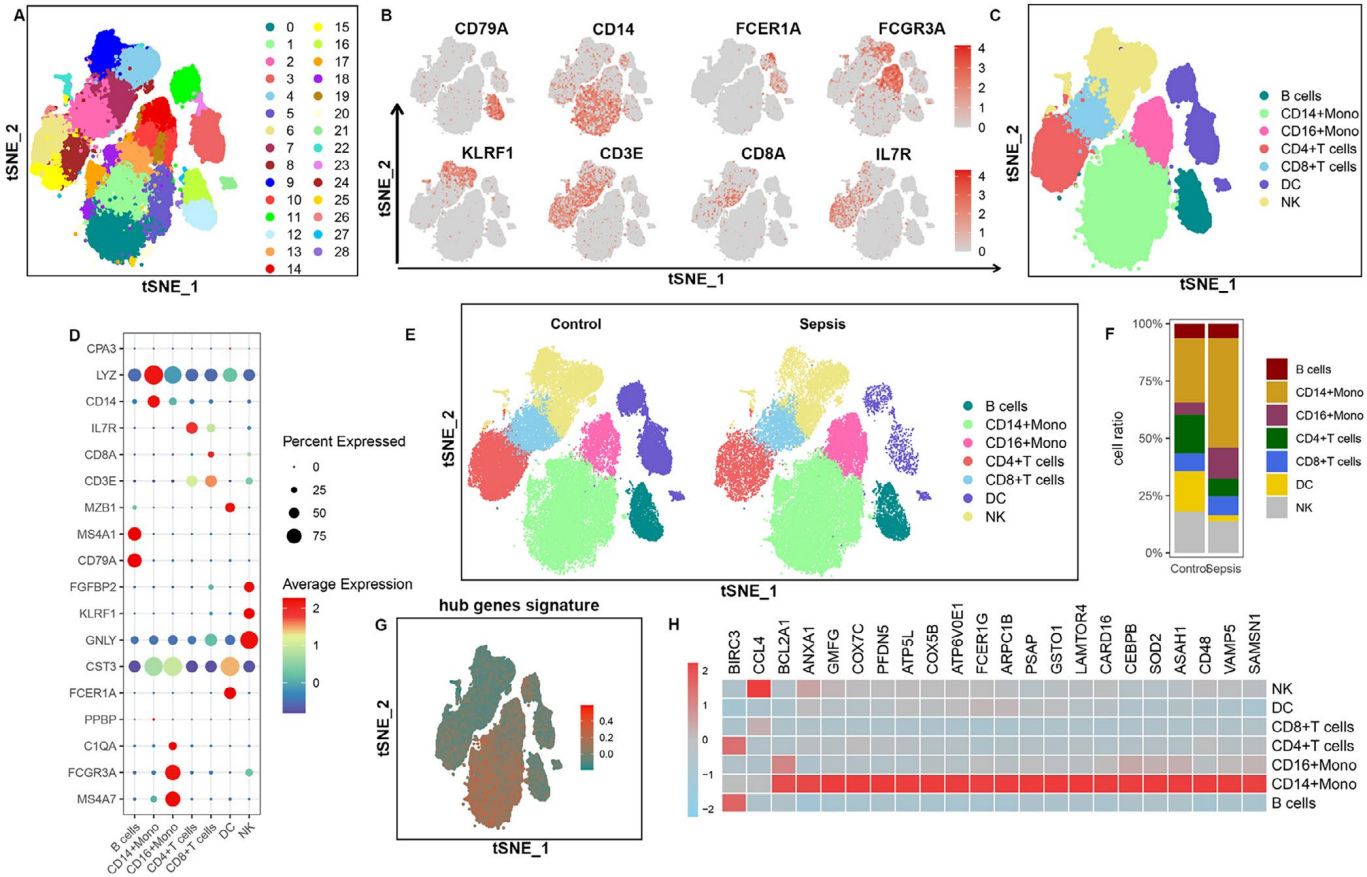
ScRNA‐seq reveals the immunological properties of DEGs in sepsis immune cell subsets. (A) tSNE plot visualises each cluster, coloured by different clusters. (B) tSNE plot showing the expression of marker genes in different clusters. (C) The tSNE plot shows each cell type, coloured by different cell types. (D) Dot plot showing the expression level of known marker genes in different cell types. (E) tSNE plot showing each myeloid cell subgroup in different groups. (F)The bar chart shows the relative frequency of each subgroup of myeloid cells in different groups. (G) The distribution of module scores of these DEGs modules in the tSNE space was evaluated in different cell types. (H) The heatmap illustrates the expression distribution of common DEGs within each subpopulation of immune cells.

To further elucidate the impact of IBD on sepsis disease, we subsequently analysed the immune profile of these hub genes by applying standard single‐cell analysis methods in the sepsis dataset SCP548. A total of 43,294 cells were acquired following standardised data processing and quality filtering, and these cellular profiles were categorised into 29 distinct clusters based on UMAP analysis (Figure 4A). Seven primary cell categories were then identified within the PBMCs, including CD14^+^ monocytes (clusters 19, 10 and 14), CD16^+^ monocytes (clusters 19, 10 and 14), B cells (clusters 16, 12 and 27), CD4^+^ T cells (clusters 6, 8 and 15), CD8^+^ T cells (clusters 2 and 7), dendritic cells (clusters 21, 3, 23 and 11) and natural killer cells (clusters 7, 4, 9, 22, 26 and 24) using classical markers (Figure 4B–D). We observed a substantial increase in the percentage of CD14^+^ monocytes in septic patients relative to healthy controls. At the same time, the proportions of CD4^+^ T cells and dendritic cells were decreased in the sepsis cohort (Figure 4E,F and Figure S5A–G). Using the ‘UCell’ package, we then quantified the module scores of these hub genes across various cell types. We found that CD14^+^ monocytes also exhibited the highest module scores in the sepsis group, significantly surpassing those of other cell types (Figure 4G). In addition, heatmap analysis further validated that most of the hub gene mains were most highly expressed in CD14^+^ monocytes from sepsis patients and higher than the control group (Figure 4H and Figure S5H). Consequently, these findings indicated that the elevated expression of hub genes in IL1B^+^ macrophages in IBD may contribute to the progression of sepsis patients through an inflammatory factor storm.

### 4.6 Common Transcriptional Modifications IL1B ^+^ Macrophages and CD14 ^+^ Monocytes in the Context of IBD and Sepsis

**FIGURE 5:**
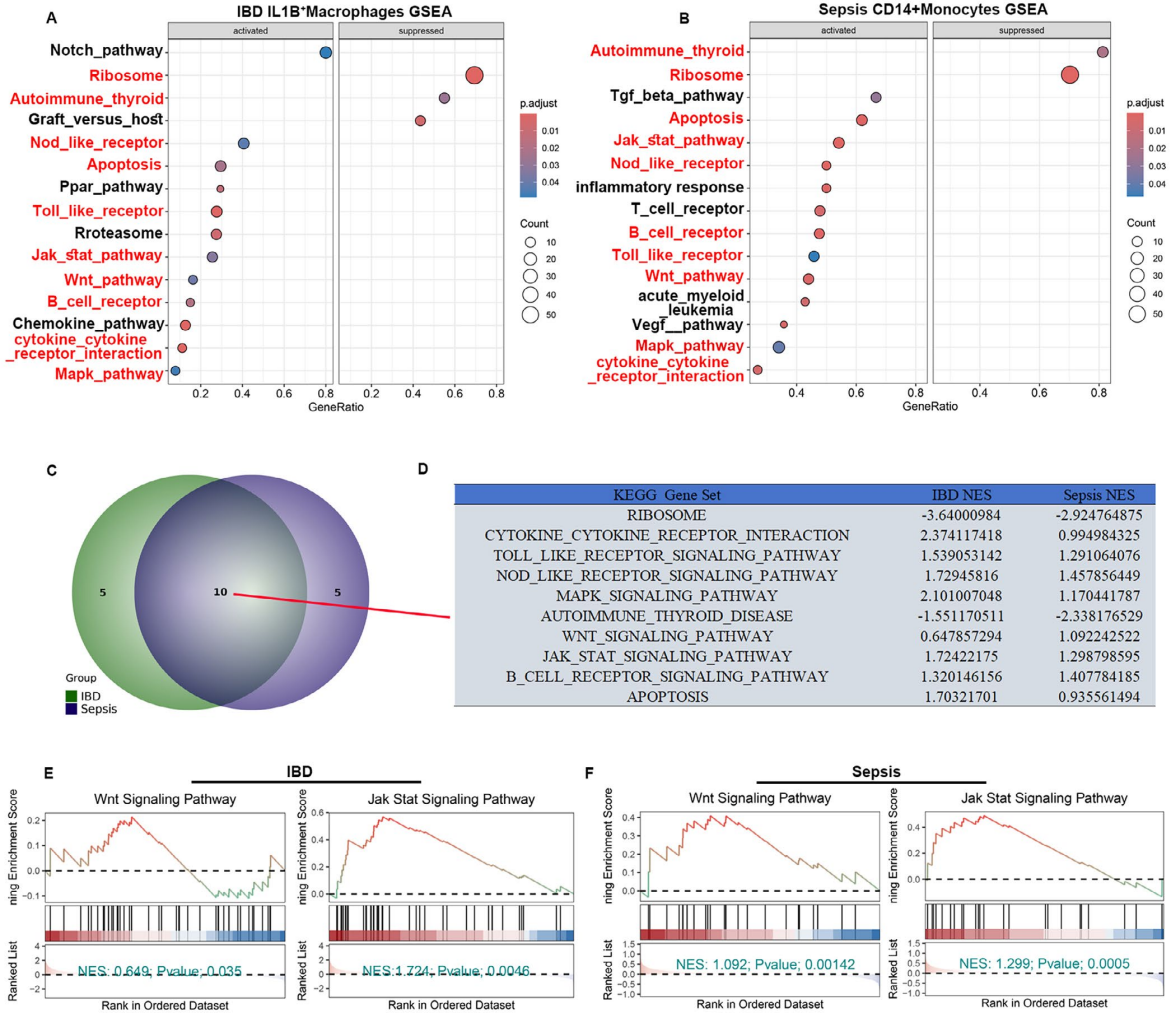
GSEA enrichment Analysis of IL1B^+^Macrophages and CD14^+^ Monocytes in IBD and Sepsis. (A) GSEA results for IL1B^+^ macrophages derived from single‐cell transcriptomic data in IBD. (B) GSEA analysis for CD14^+^monocytes derived from single‐cell transcriptomic data in Sepsis. (C, D) Venn and table diagram showing common pathways between IL1B^+^macrophages and CD14^+^monocytes bulk‐RNA data in IBD and sepsis cases. (E, F) Selected GSEA pathway analyses of IL1B^+^macrophages and CD14^+^monocytes for IBD and Sepsis.

We investigated possible IL1B^+^macrophages and CD14^+^ monocyte pathways since systemic IBD and Sepsis are associated with enhanced cell ratios and pronounced hub gene expression. Six hundred thirty‐five upregulated genes and 290 down‐regulated genes were obtained by performing DEGs on IL1B^+^ macrophages from IBD and control groups (Figure S6A). For Sepsis, 250 DEGs, including 147 upregulated genes and 103 downregulated genes within CD14^+^ monocytes, were identified when sepsis patients were compared to healthy controls (Figure S6C). We performed functional pathway enrichment analysis on these DEGs of IL1B^+^ macrophages and CD14^+^ monocytes and found that these DEGs were commonly enriched in inflammatory response‐related pathways, such as the IL‐17 signalling pathway, TNF and JAK–STAT signalling pathway (Figure S6B,D). Moreover, GSEA enrichment was further performed for these genes in IL1B^+^ macrophages and CD14^+^ monocytes. Interestingly, 10 of the 15 activated pathways found in Sepsis were also activated in IBD, particularly pathways like Wnt, NOD‐like receptor and Toll‐like receptor pathway (Figure 5A–D). Furthermore, normalised enrichment scores (NES) indicated that these pathways were active in both conditions (Figure 5E,F and Figure S6E,F). Therefore, these converging pathways advance our understanding of the possible molecular associations between IBD and Sepsis, particularly immune‐related signalling pathways.

### 4.7 Significant Diagnostic Value of BCL2A1 and CEBPB in Sepsis

**FIGURE 6:**
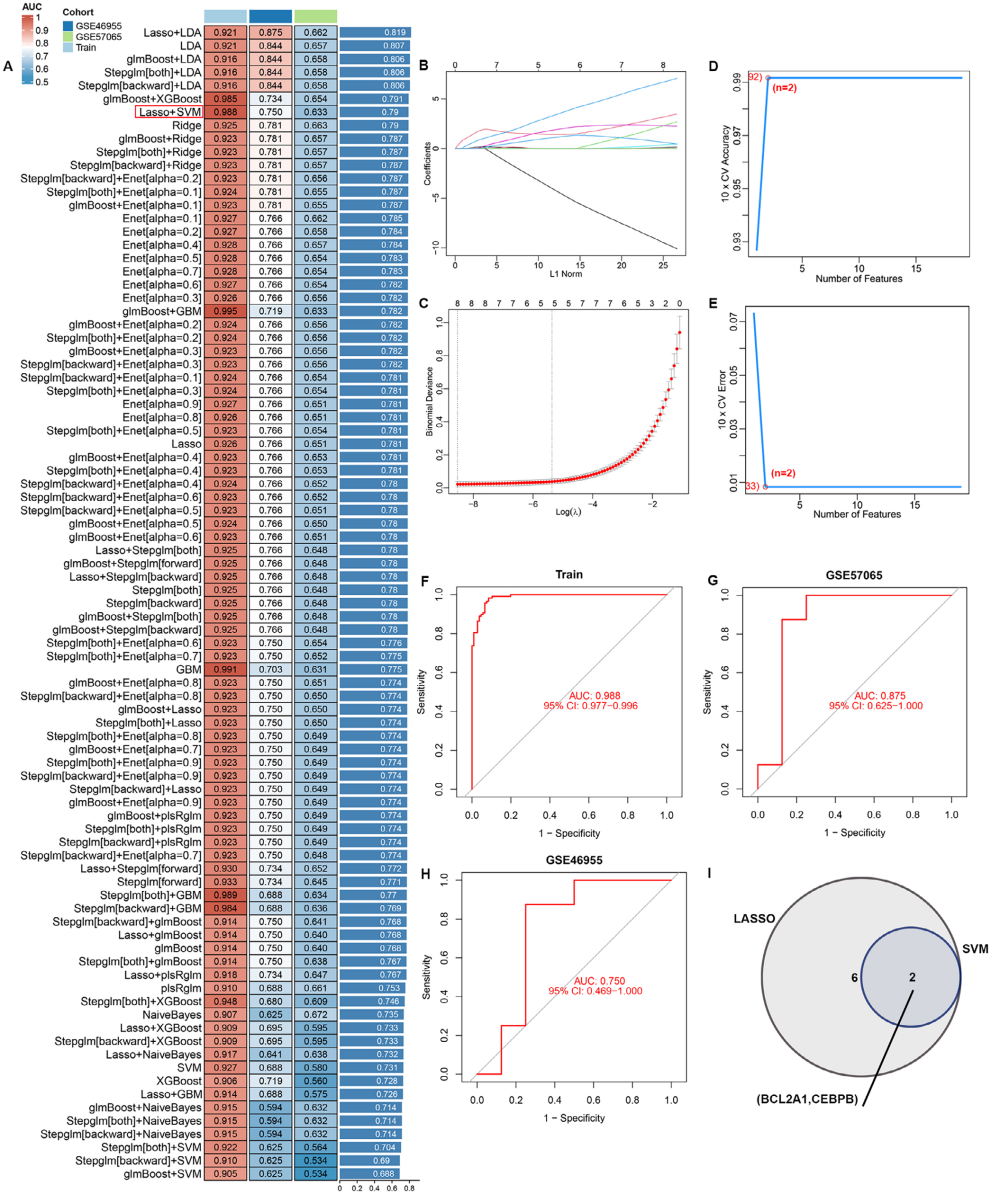
Screening candidate diagnostic biomarkers for MS by machine learning. (A) C‐index of multiple models derived from combinations of different machine learning algorithms in three cohorts. (B) LASSO regression for the predictive genes, the plot illustrates the Lasso coefficients for each gene in relation to the logarithmically scaled λ value. (C) Penalty plot of cross‐validated Mean Square Error (MSE) for the Lasso model. (D, E) Support Vector Machine‐Recursive Feature Elimination (SVM‐RFE) algorithm for screening feature genes. (F–H) ROC curve analysis of the C index of the Lasso joint SVM over time in multiple cohorts. (I) Venn diagram illustrates that two candidate diagnostic genes are identified via the two algorithms.

To develop a robust predictive model, we used selected genes as input features and evaluated 10 machine learning methods: Random Survival Forest (RSF), Elastic Net (Enet), stepwise Cox, CoxBoost, Partial Least Squares Regression for Cox (plsRcox), Lasso, Ridge, Supervised Principal Components (SuperPC), Gradient Boosting Machine (GBM) and survival‐SVM. Using the dataset GSE95233 as test data alongside two external validation datasets (GSE57065 and GSE46955), we compared the concordance index (C‐index) of these models. Lasso combined with SVM emerged as the predominant model due to its superior C‐index (Figure 6A). An optimal diagnostic signature was then developed using the integrated Lasso and SVM algorithms (Figure 6B–E). We verified the model's performance through Receiver Operating Characteristic (ROC) analysis. For the training sepsis cohort, the Area under the curve (AUC) was 0.988; the test datasets GSE57065 and GSE46955 exhibited AUCs of 0.875 and 0.750, respectively (Figure 6F–H). The top two important features identified among these 23 hub genes using these algorithms were BCL2A1 and CEBPB (Figure 6I).

### 4.8 Validation of BCL2A1 and CEBPB in Mice With IBD Combined With Sepsis

**FIGURE 7:**
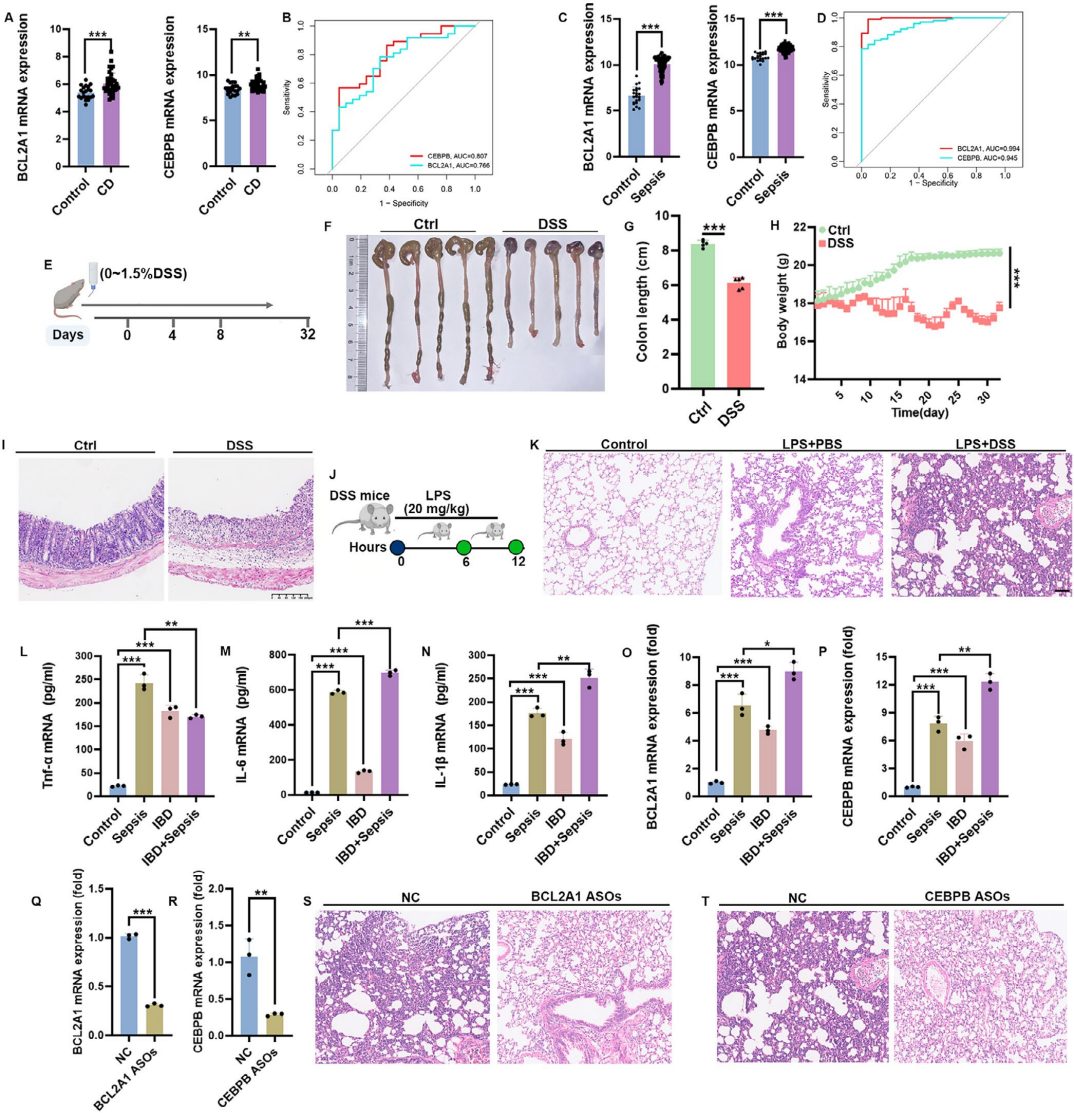
Real‐Time PCR Confirms Elevated BCL2A1 and CEBPB mRNA Levels in Mice with IBD Combined with Sepsis. (A) The difference in mRNA expression levels of BCL2A1 and CEBPB in IBD patients based on datasets GSE126124 and GSE9686. (B) ROC curve analysis of BCL2A1 and CEBPB in IBD patients based on datasets GSE126124 and GSE9686. (C) The difference in mRNA expression of BCL2A1 and CEBPB in sepsis patients based on the GSE95233 cohort. (D) ROC curve analysis of BCL2A1 and CEBPB in sepsis patients based on GSE95233 cohort. (E) Diagrammatic representation of mice that were fed DSS. (F, G) Representative images of the colon are then compared for length. *n* = 5. (H) Changes in body weight of mice. (I) Representative H&E‐stained colon. Scale bar = 200 μm. (J) Diagrammatic representation of IBD mice that were fed LPS. (K) Representative H&E‐stained colon. Scale bar = 200 μm. (L–N) The results of the ELISA tests confirmed the levels of inflammation factors (Tnf‐α, Il‐6 and Il‐1β) in mouse serum from control, Sepsis, IBD and IBD complicated by Sepsis. (O–P) The results of the qPCR tests confirmed the mRNA levels of BCL2A1 and CEBPB in mouse PBMCs from control, Sepsis, IBD and IBD complicated by Sepsis. (Q–R) The results of the qPCRs tests confirmed the mRNA levels of BCL2A1 and CEBPB in intestinal tissues from sepsis treated with BCL2A1 ASOs or CEBPB ASOs. (S–T) Representative images of the lung tissues in sepsis mice treated with BCL2A1 ASOs or CEBPB ASOs. *p < 0.05; **p < 0.01; ***p < 0.001.

To explore the mRNA expression levels of BCL2A1 and CEBPB, we consulted datasets from healthy individuals as well as patients with Sepsis and IBD. The expression level of these two genes was significantly upregulated in patients compared with controls (Figure 7A,C and Figure S7A). Additionally, the ROC analysis confirmed that the expression of BCL2A1 and CEBPB were of great diagnostic value in IBD and Sepsis (Figure 7B,D) and (Figure S7B). Meanwhile, additional datasets (GSE75214, GSE57065 and GSE46955) further confirmed the high expression of these two genes in both IBD (Figure S7C,D) and Sepsis (Figure S7E–H). Subsequently, we induced IBD in mice using DSS following established protocols (Figure 7E). We observed a marked decrease in body weight in DSS‐treated mice compared to controls, coupled with a significant reduction in colon length (Figure 7F–H). Histological analysis revealed severe mucosal damage in DSS‐treated mice, characterised by marked villous degeneration (Figure 7I). After inducing the LPS sepsis model for 12 h (Figure 7J), we discovered that IBD dramatically increased sepsis‐associated lung damage and inflammatory expression compared to controls, indicating that IBD can promote sepsis progression (Figure 7K–N). To confirm the mRNA expression levels of BCL2A1 and CEBPB, we examined PBMCs extracted from these murine models. BCL2A1 and CEBPB expression levels were significantly increased in mice with Sepsis complicated by IBD (Figure 7O,P). Antisense oligonucleotides (ASOs) potentially silence therapeutic targets. Therefore, we hypothesise that BCL2A1 ASOs or CEBPB ASOs can influence the progression of Sepsis by targeting BCL2A1 and CEBPB in the intestines of mice. Firstly, we subsequently intrarectally injected cholesterol‐conjugated CL2A1 ASOs or CEBPB ASOs for 2 weeks and observed that BCL2A1 ASOs or CEBPB ASOs, as opposed to the negative control (NC) ASOs, effectively reduced the mRNA levels of BCL2A1 and CEBPB in intestinal tissues (Figure 7Q,R). Importantly, BCL2A1 ASOs or CEBPB ASOs mitigated lung damage in septic mice compared to controls (Figure 7S,T). These observations reinforce our preliminary findings and suggest that BCL2A1 and CEBPB may play a subtle role in the pathologic process of Sepsis exacerbated by IBD.

## 5 Discussion

Sepsis is a systemic inflammatory response syndrome associated with a high mortality rate. The pathogenesis of Sepsis is complex and remains poorly elucidated. A persistent exaggerated inflammatory response coupled with immune suppression are key aspects of sepsis pathophysiology [34]. IBD increases the secretion of pro‐inflammatory cytokines, including TNF‐α and chemokines [35, 36]. These factors may lead to the excessive activation of the innate immune system [37]. Recent studies suggest associations among various diseases, highlighting the importance of exploring disease interconnections—a promising field for future research [38, 39], such as extracellular vesicles [40, 41, 42, 43, 44, 45, 46, 47], inflammation factors or genetic modification. In this study, we investigate the potential interactions between IBD and Sepsis from a multi‐omics perspective. We aim to elucidate the interplay between IBD and Sepsis, offering new directions and potential targets for treating these diseases.

In this study, the analysis of IBD and sepsis transcriptomics revealed that 23 shared hub DEGs were identified in both conditions. Several hub genes have been confirmed to play key roles in immune function, including inflammation and immunomodulation. To determine the all‐immune cell type responsible for the expression of these hub DEGs, we further investigated the characterisation of single‐cell profiles of IBD and sepsis samples. We emphasised that the number of myeloid cells (IL1B^+^ macrophages or CD14^+^ monocytes) significantly increased in both conditions, and most hub genes are enriched in these cell types, with expression levels higher than those in the control group. Studies have confirmed that the disruption of colonic homeostasis induced by M1/M2 macrophage polarisation contributes to the development of IBD [48]. Regarding innate immunity, the number of circulating monocytes (CD14^+^ monocytes) is increased in patients with Sepsis, and the monocytic distribution width has emerged as a promising biomarker for Sepsis [49]. Here, we found that these DEGs are primarily enriched in IL1B^+^ macrophages or CD14^+^ monocytes from IBD and sepsis patients. Therefore, we can infer that these hub genes, such as Il1b, S100a, Il1rn and other inflammatory factors, may promote the transformation of macrophages into a pro‐inflammatory phenotype, triggering the major causes of cytokine storms. This also suggests that in IBD patients, the induced macrophage inflammatory response may contribute to the progression of Sepsis. Therefore, this evidence indicates a role for myeloid cells (IL1B^+^ macrophages or CD14^+^ monocytes) as a shared cellular associated with the progression of Sepsis and IBD.

To further dissect the potential mechanisms of immune dysregulation common to IBD and Sepsis, we performed a comprehensive functional pathway enrichment analysis of monocyte/macrophage transcriptomic data. Interestingly, there is a significant overlap in the pathways of aberrant activation observed in macrophages from IBD patients and monocytes from sepsis patients, including the IL‐17 signalling pathway, complement activation and TNF signalling pathway. Studies have shown that elevated complement activation is associated with tissue inflammation and damage in IBD [50] while also playing a role in the immune hyperactivation response of Sepsis. Inflammation response pathways, such as the L‐17 signalling pathway, were hyperactivated in IBD and regulated intestinal Inflammation by inducing intestinal epithelial damage [51]. Similarly, the IL‐17 signalling pathway promotes cellular pyroptosis in pneumonia‐induced Sepsis through activation of NLRP3 inflammatory vesicles [52]. Moreover, as a facilitator of inflammation diseases, TNF‐α regulates both conditions by mediating signalling pathways such as NF‐κB [53] and AKT [54]. In the mouse model of IBD combined with Sepsis, we confirmed that IBD promotes the progression of Sepsis. Therefore, the co‐activation of these critical pathways may exacerbate the inflammatory state in IBD, making patients more susceptible to complications such as Sepsis.

Consistently, we screened out two hub genes as candidate biomarkers of IBD and Sepsis, using Lasso and NaiveBayes machine learning. BCL2A1 and CEBPB were identified as potential biomarkers with diagnostic value of IBD and Sepsis. These two genes are significantly upregulated in both IBD and sepsis diseases and are highly expressed in macrophages/monocytes compared to other cell subpopulations. Moreover, in a mouse model of IBD combined with Sepsis, these two genes were found to be significantly elevated in IBD combined with sepsis mice compared to controls. Among them, the BCL2A1 gene is a critical regulator of cell death, which is a member of the B‐cell lymphoma 2 (BCL2) protein family [55]. BCL2A1 has already been [56] regarded as a highly regulated nuclear factor κB (NF‐κB) target gene [55], and it is also upregulated in Sepsis patients [57]. NF‐κB activation can mediate sepsis development [58]. BCL2A1 may, therefore, induce sepsis progression by mediating NF‐κB activation. CEBPB (CCAAT/enhancer‐binding protein beta) is essential for inflammation and is frequently activated by inflammatory mediators, such as anabolic growth hormones and immunoregulatory cytokines [59]. In addition, CEBPB has recently been identified as a key immune‐related gene in sepsis patients [60] and a potential therapeutic target for the prevention of liver failure in sepsis patients [61]. Cumulatively, these findings indicated that there are common cellular and potential molecular mechanisms between IBD and Sepsis, which may have significant implications for disease progression.

Despite the advances our study contributes to a deeper understanding of IBD and Sepsis, there are several limitations. First, our reliance on publicly available datasets may not fully capture the heterogeneity of these conditions. Additionally, our findings are primarily based on bioinformatics analyses, which necessitate further experimental validation. While our research highlights potential cellular connections and pathway alterations, the specific molecular mechanisms remain to be elucidated and validated, for example, whether the pro‐inflammatory transformation of intestinal macrophages affects the onset of Sepsis and whether the activation of the IL‐17 and TNF signalling pathways triggers the progression of Sepsis. Finally, it remains to be explored whether BCL2A1 and CEBPB can be incorporated into existing diagnostic protocols as potential biomarkers for both IBD and Sepsis, with the aim of improving disease detection and prognosis. Further validation of the expression levels of these biomarkers across different patient populations is necessary. This will help ensure their applicability in diverse clinical settings, ultimately contributing to improved patient outcomes.

## 6 Conclusions

To conclude, this study is the first to explore the potential molecular and cellular intersections between IBD and Sepsis through a comprehensive analysis of transcriptomics, single‐cell transcriptomics and experimental data. This research demonstrates the impact of IBD on the progression of Sepsis and analyses two DEGs, BCL2A1 and CEBPB, as potential diagnostic biomarkers for both IBD and Sepsis.

## Author Contributions

**Chao Liu:** conceptualization (lead), writing – original draft (equal). **Jinliang Liu:** writing – review and editing (equal). **Yitian Yang:** writing – review and editing (equal).

## Conflicts of Interest

The authors state that the research was conducted without any commercial or financial relationships that might be interpreted as potential conflicts of interest.

## Supporting information


Data S1.

## Data Availability

All data supporting the results of this study can be obtained from the corresponding author upon reasonable request.
