# Supplementary material for: Bulk and Single‐Cell Transcriptomic Reveals Shared Key Genes and Patterns of Immune Dysregulation in Both Intestinal Inflammatory Disease and Sepsis
Source: J Cell Mol Med. 2025 Feb 24;29(4):e70415. doi: 10.1111/jcmm.70415 (PMC11850196; doi:10.1111/jcmm.70415)
Supplement: Supplementary file 1 — Data S1. [file JCMM-29-e70415-s001.docx]

**
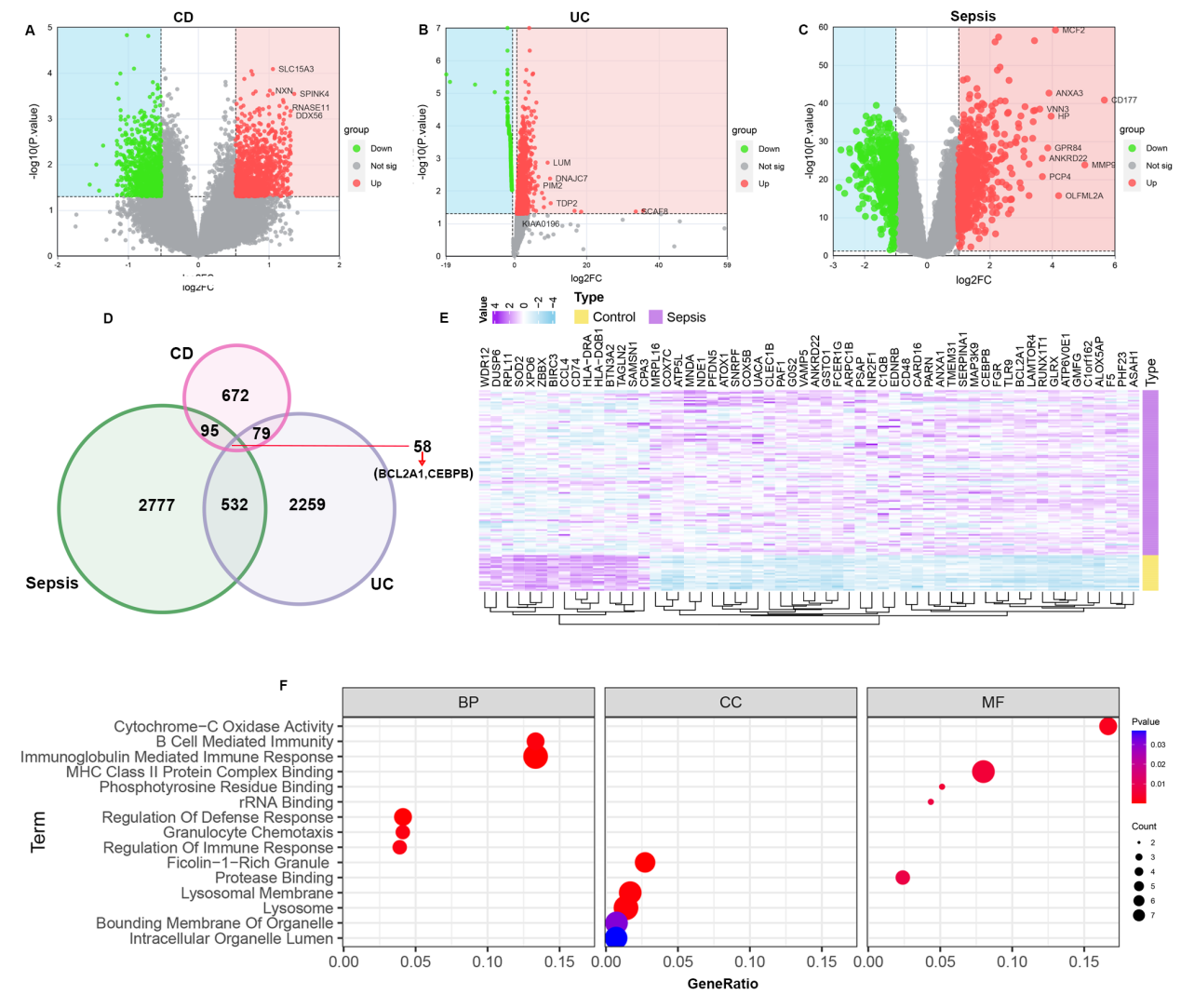
**

**Supplementary Figure.s1. Identifification of shared DEGs between IBD and Sepsis.**

1. C) The volcano plot of DEGs in IBD (including CD and UC) and Sepsis, pink and green represent significant DEG. (D) The Venn diagrams showed that 58 common DEGs between IBD and sepsis datasets. (E) The heatmap displays the top 30 DEGs from IBD and sepsis datasets. (F) Gene Ontology (GO) enrichment analysis for the shared DEGs.


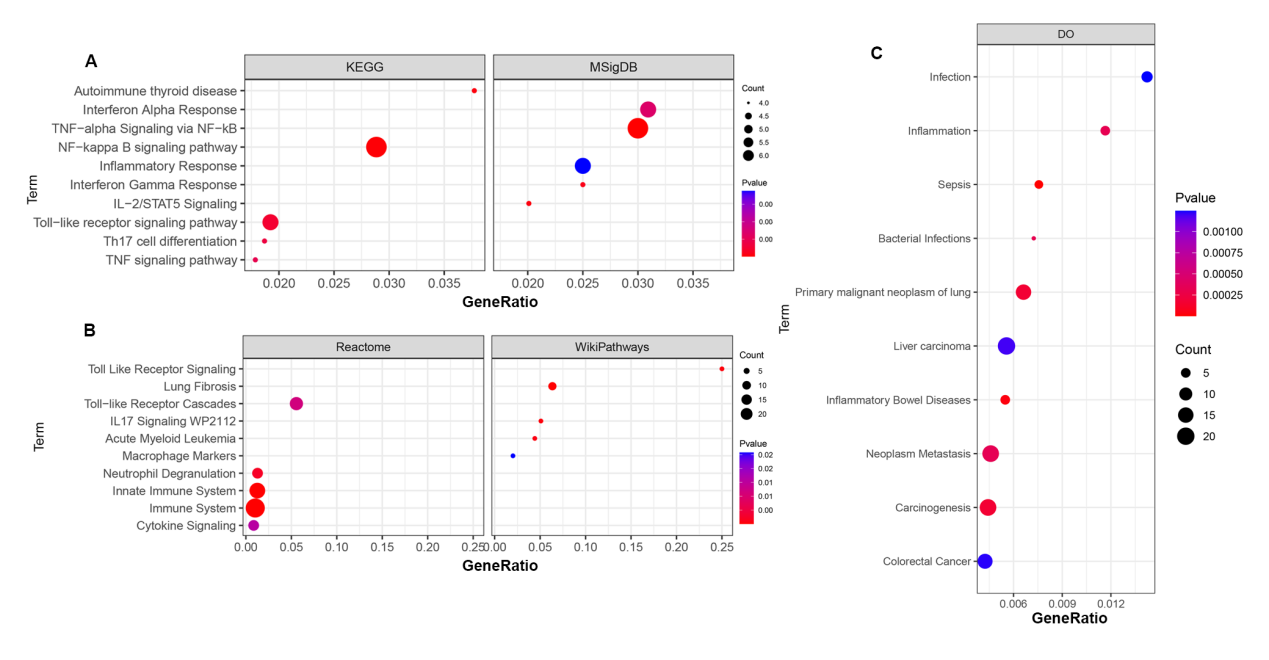


**Supplementary Figure.s2. Pathway enrichment analysis of shared DEGs between IBD and Sepsis.**

1. B) KEGG and MSigDB Pathways enrichenmet analysis of common DEGs from IBD and sepsis datasets. (B) Reactome and WikiPathway pathway enrichment of common DEGs from IBD and sepsis datasets. (C) DO enrichemnt analysis ofshared DEGs from IBD and sepsis datasets.


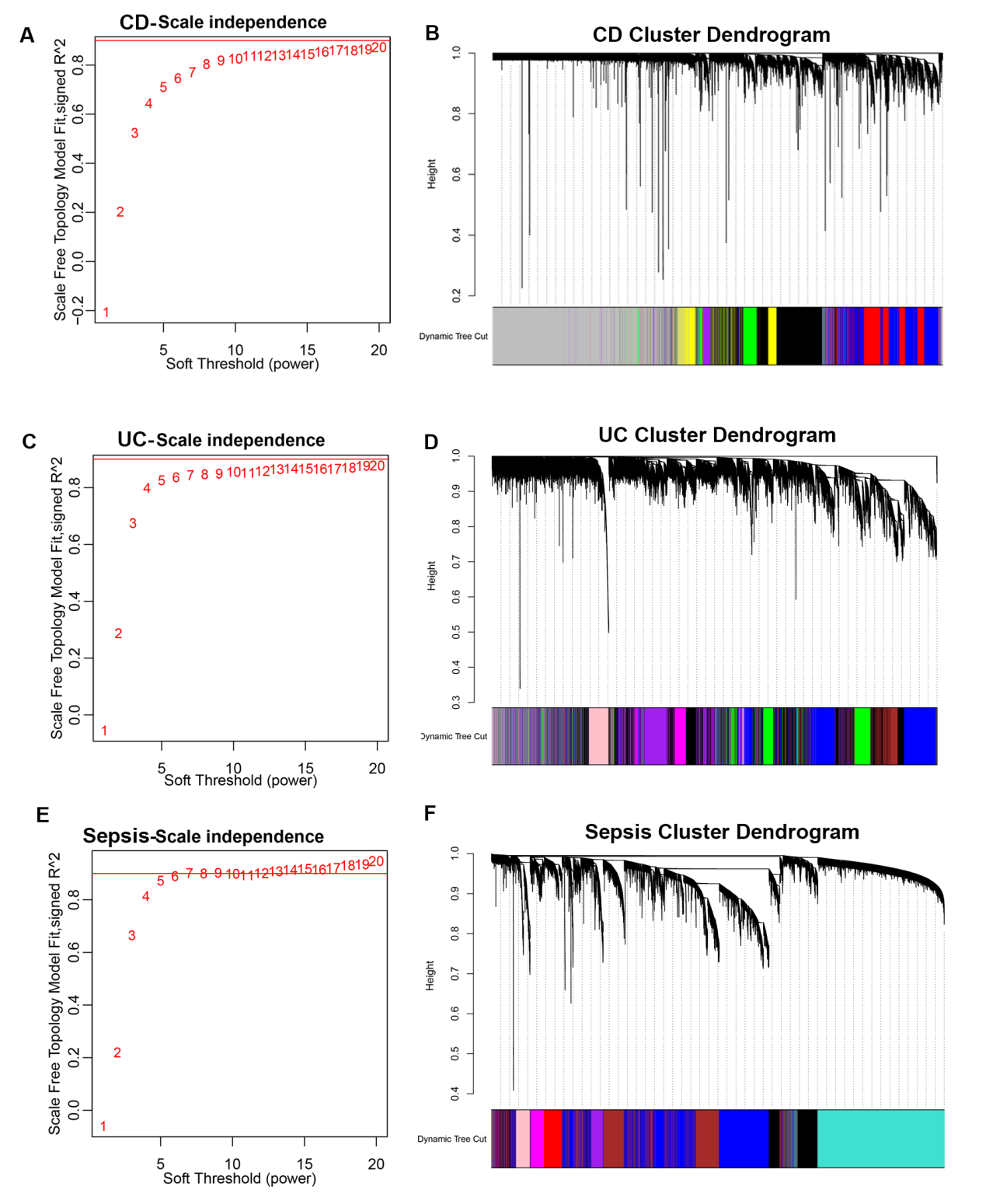


**Supplementary Figure.s3. Selection of soft threshold for WGCNA analysis.**

(A, C, E) Clustering dendrogram of genes in UC, CD and Sepsis cohorts, various colors represent different modules. (B, D, F) Choosing the best soft-threshold power in CD, UC, and Sepsis cohorts.


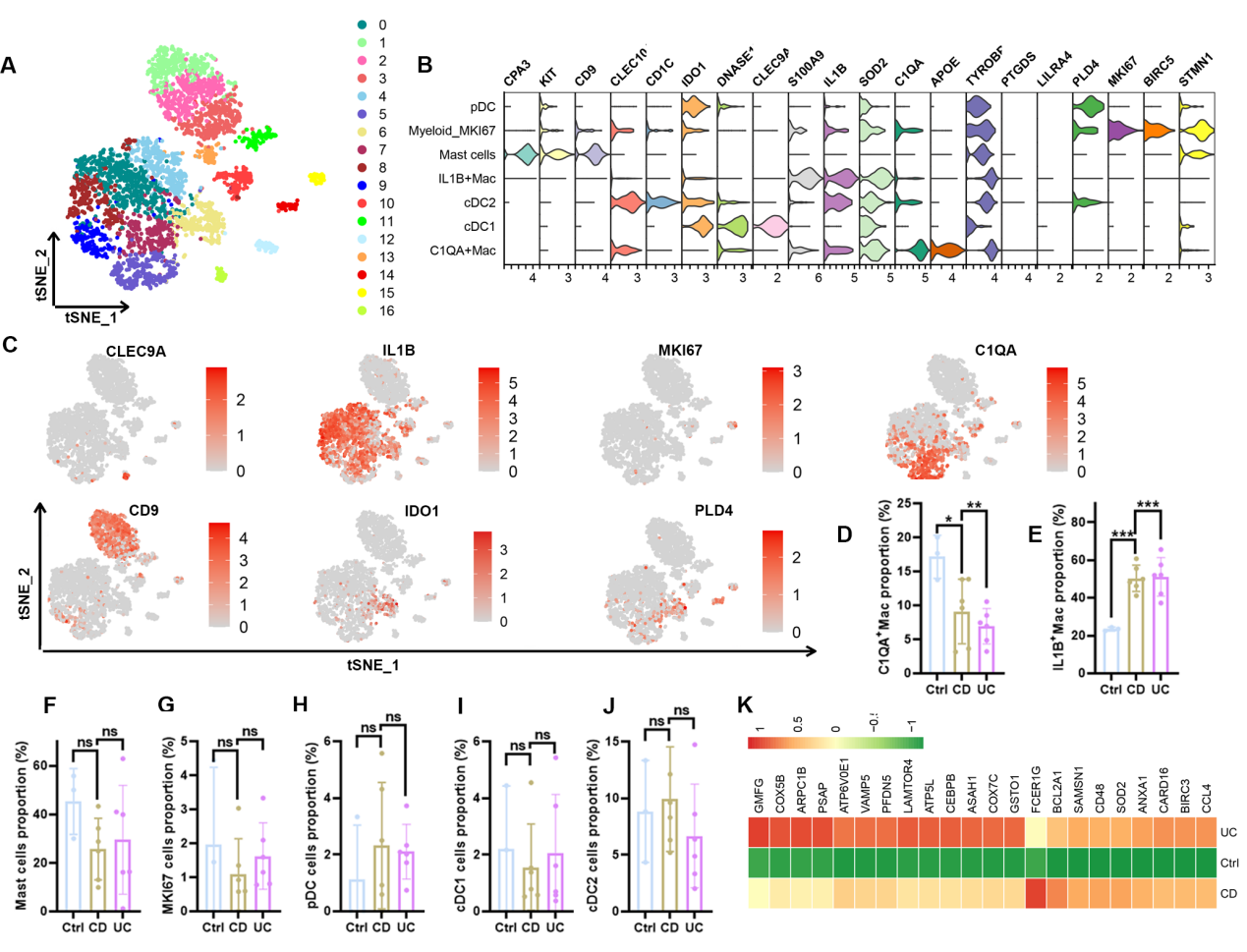


**SupplementaryFigure.s4.tSNE plot illustrating marker gene expression in different cell types.**

1. tSNE plot visualizing 17 clusters of myeloid cells between IBD (UC and CD) and control samples. (B) The violin plot shows the expression of the marker genes in different cell types. (C) tSNE plot showing the expression of marker genes in different clusters. (D-J) Proportion of myeloid cell subgroups between IBD and control samples. (K) The heatmap displays the expression patterns of DEGs among CD, UC and control samples.


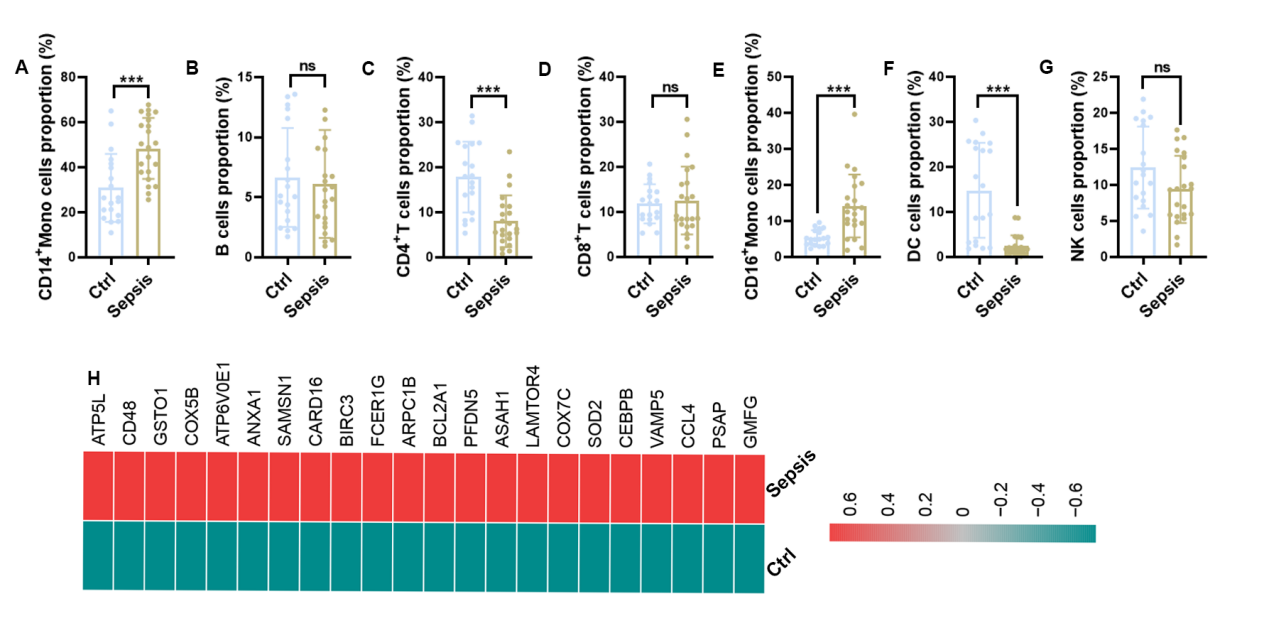


**Supplementary Figure.s5. Proportion of the different immune cell subgroups in sepsis single cell dataset.**

1. G) Proportion of immune cell subgroups between sepsis and control samples. (H) The heatmap displays the expression patterns of DEGs between sepsis and control samples.


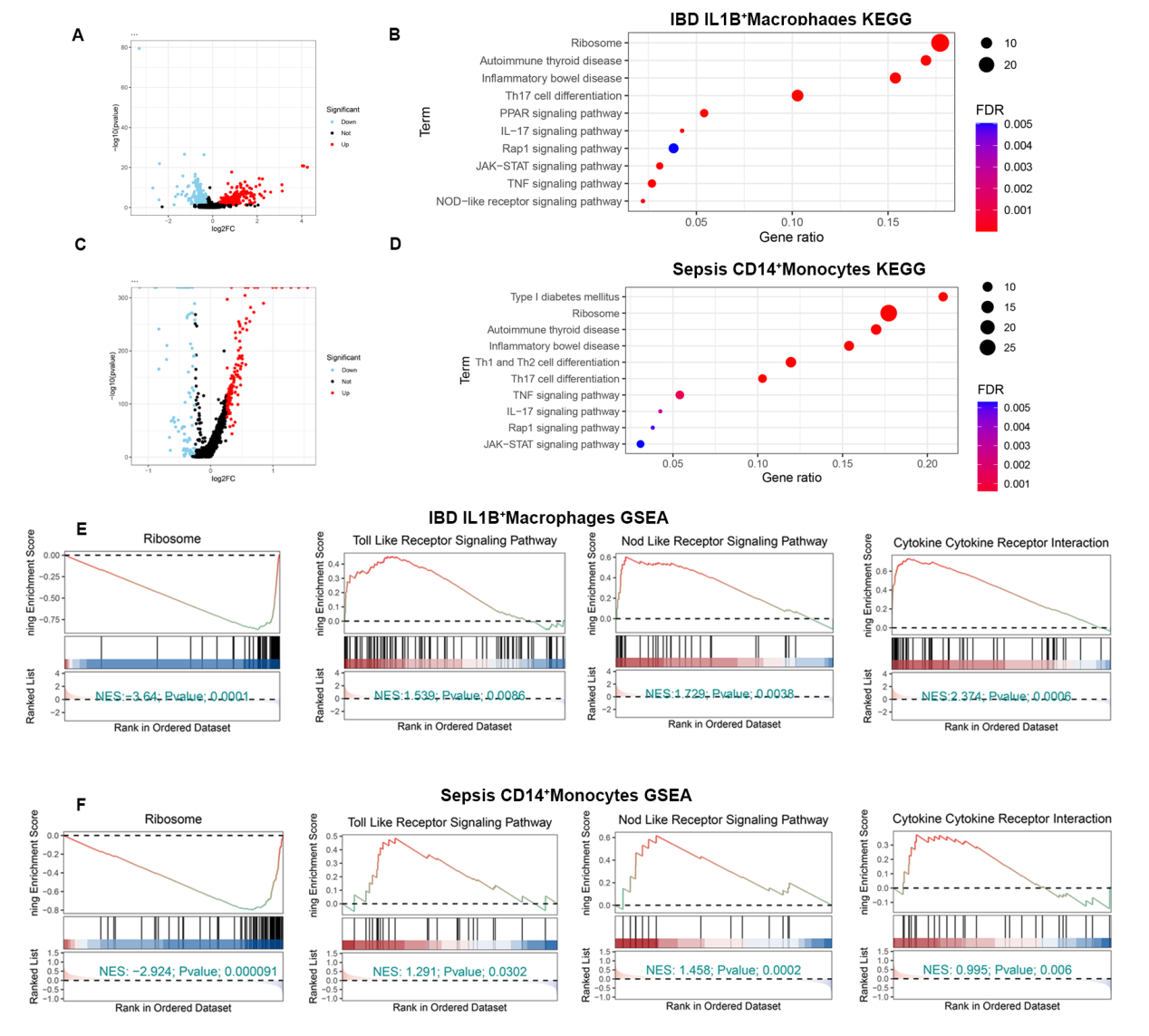


**Supplementary Figure.s6. GSEA enrichment analysis of IL1B^+^macrophages and CD14^+^ Monocytes in IBD and Sepsis.**

1. Volcano plot of DEGs in IL1B^+^ macrophages from IBD patients and healthy controls. (B)Top 10 enriched pathways in KEGG analysis of IL1B^+^ macrophages from IBD and control samples.(C)Volcano plot of DEGs in CD14^+^ monocytes from sepsis patients and healthy controls.(D)Top 10 enriched pathways in KEGG analysis of CD14^+^ monocytes from IBD and control samples. (E)GSEA outcomes for IL1B^+^macrophages derived from single-cell transcriptomic data in IBD. (F) The results of GSEA for CD14^+^ monocytes derived from single-cell transcriptomic data in sepsis.


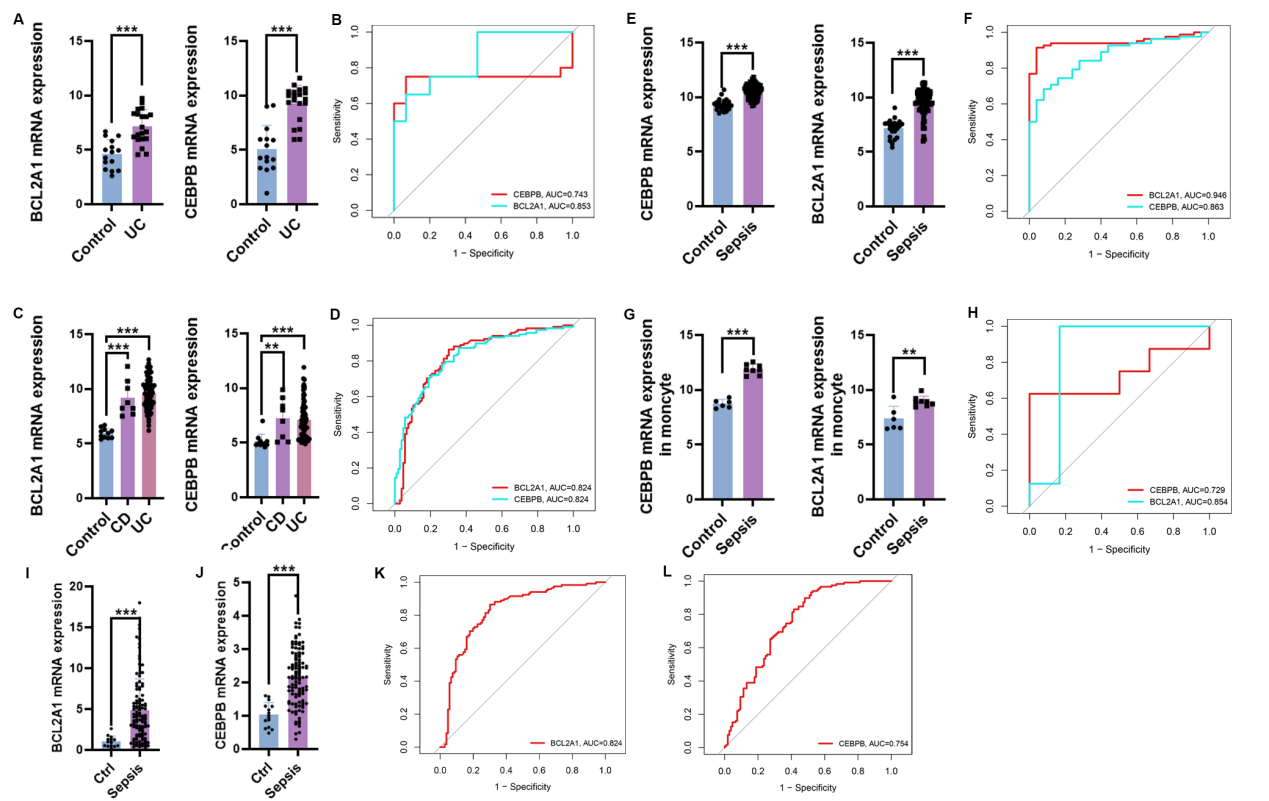


**Supplementary Figure.s7. Verification the expression and ROC curve for the BCL2A1 and CEBPB in IBD and sepsis patients from the verification cohort.**

1. The difference in mRNA expression levels of BCL2A1 and CEBPB in UC patients, based on dataset GSE75214. (B) ROC curve results for BCL2A1 and CEBPB in UC patients, based on dataset GSE75214. (C) The difference in mRNA expression levels of BCL2A1 and CEBPB in IBD and control samples, based on dataset GSE75214. (D) ROC curve results for BCL2A1 and CEBPB in IBD and control samples, based on dataset GSE46955. (E) The results of the mRNA expression level for BCL2A1 and CEBPB in sepsis patients, based on dataset GSE57065. (F) The results of ROC curve for BCL2A1 and CEBPB in sepsis and control samples, based on dataset GSE57065. (G) The mRNA expression of BCL2A1 and CEBPB in human monocytes from SLE and control, obtained from dataset GSE46955. (H) The results of ROC curve for BCL2A1 and CEBPB in human monocytes from SLE and control, obtained from dataset GSE46955.(I-J) The mRNA expression of BCL2A1 and CEBPB in Sepsis and control, obtained from dataset GSE4607. (K-L) The results of ROC curve for BCL2A1 and CEBPB in Sepsis and control, obtained from dataset GSE4607.

**Supplementary Tables**

**Supplementary Table1. Primer for BCL2A1 and CEBPB.**

| **BCL2A1** | **Forward Sequence** | **GGATAAGGCAAAACGGAGGCTG** |
| --- | --- | --- |
|  | **Reverse Sequence** | **CAGTATTGCTTCAGGAGAGATAGC** |
| **CEBPB** |  |  |
|  | **Forward Sequence** | **AGAAGACCGTGGACAAGCACAG** |
|  | **Reverse Sequence** | **CTCCAGGACCTTGTGCTGCGT** |

**Supplementary Table2. Supplementary Table 2. The top 5 enriched GO terms of the shared DEGs.**

| **Term** | **ONTOLOGY** | **Pvalue** | **Count** | **GeneRatio** |
| --- | --- | --- | --- | --- |
| **Immunoglobulin Mediated Immune Response** | **BP** | **8.89E-05** | **7** | **0.133333333** |
| **Regulation Of Defense Response** | **BP** | **1.80E-04** | **4** | **0.041237113** |
| **B Cell Mediated Immunity** | **BP** | **8.47E-04** | **4** | **0.133333333** |
| **Granulocyte Chemotaxis** | **BP** | **0.001246406** | **3** | **0.04109589** |
| **Regulation Of Immune Response** | **BP** | **0.001453954** | **3** | **0.038961039** |
| **Lysosome** | **CC** | **6.05E-04** | **7** | **0.013916501** |
| **Lysosomal Membrane** | **CC** | **5.66E-04** | **6** | **0.016853933** |
| **Bounding Membrane Of Organelle** | **CC** | **0.030802389** | **6** | **0.007326007** |
| **Intracellular Organelle Lumen** | **CC** | **0.037040987** | **6** | **0.007009346** |
| **Ficolin-1-Rich Granule** | **CC** | **1.93E-04** | **5** | **0.027173913** |
| **Cytochrome-C Oxidase Activity** | **MF** | **0.002375491** | **4** | **0.166666667** |
| **MHC Class II Protein Complex Binding** | **MF** | **0.00571686** | **6** | **0.08** |
| **Phosphotyrosine Residue Binding** | **MF** | **0.005725432** | **2** | **0.051282051** |
| **Protease Binding** | **MF** | **0.007882153** | **3** | **0.024** |
| **rRNA Binding** | **MF** | **0.00755343** | **2** | **0.043478261** |

**Supplementary Table3. Supplementary Table 3. The top 10 enriched D0 terms of the shared DEGs.**

| **Term** | **ONTOLOGY** | **Pvalue** | **Count** | **GeneRatio** |
| --- | --- | --- | --- | --- |
| **Primary malignant neoplasm of lung** | **DO** | **0.000168** | **15** | **0.006613757** |
| **Carcinogenesis** | **DO** | **0.000171** | **18** | **0.004428044** |
| **Bacterial Infections** | **DO** | **0.00032** | **2** | **0.007246377** |
| **Inflammation** | **DO** | **0.000324** | **5** | **0.011655012** |
| **Neoplasm Metastasis** | **DO** | **0.000335** | **18** | **0.004591837** |
| **Liver carcinoma** | **DO** | **0.001197967** | **20** | **0.005566379** |
| **Colorectal Cancer** | **DO** | **0.001227386** | **14** | **0.004244997** |
| **Infection** | **DO** | **0.001246075** | **7** | **0.014227642** |
| **Sepsis** | **DO** | **5.72E-09** | **4** | **0.007561437** |
| **Inflammatory Bowel Diseases** | **DO** | **0.000032** | **5** | **0.005488474** |

**Supplementary Table4. Supplementary Table 4. Enriched KEGG Pathwas and MSigDB associated with the shared DEGs.**

| **Term** | **ONTOLOGY** | **Pvalue** | **Count** | **GeneRatio** |
| --- | --- | --- | --- | --- |
| **NF-kappa B signaling pathway** | **KEGG** | **9.82E-05** | **6** | **0.028846154** |
| **Autoimmune thyroid disease** | **KEGG** | **2.35E-04** | **4** | **0.037735849** |
| **Toll-like receptor pathway** | **KEGG** | **9.72E-04** | **5** | **0.019230769** |
| **Th17 cell differentiation** | **KEGG** | **0.001509016** | **4** | **0.018691589** |
| **TNF signaling pathway** | **KEGG** | **0.001931274** | **4** | **0.017857143** |
| **TNF-alpha Signaling via NF-kB** | **MSigDB** | **2.44E-05** | **6** | **0.03** |
| **IL-2/STAT5 Signaling** | **MSigDB** | **2.83E-04** | **4** | **0.020100503** |
| **Interferon Gamma Response** | **MSigDB** | **3.88E-04** | **4** | **0.025** |
| **Interferon Alpha Response** | **MSigDB** | **0.002700856** | **5** | **0.030927835** |
| **Inflammatory Response** | **MSigDB** | **0.007519809** | **5** | **0.025** |

**Supplementary Table4. Supplementary Table 5. Enriched Reactome Pathwas and WikiPathways associated with the shared DEGs.**

| **Term** | **ONTOLOGY** | **Pvalue** | **Count** | **GeneRatio** |
| --- | --- | --- | --- | --- |
| **Immune System** | **Reactome** | **2.49E-07** | **20** | **0.010293361** |
| **Innate Immune System** | **Reactome** | **6.35E-06** | **13** | **0.012560386** |
| **Toll-like Receptor Cascades** | **Reactome** | **0.011623478** | **9** | **0.055555556** |
| **Neutrophil Degranulation** | **Reactome** | **0.002300294** | **6** | **0.012820513** |
| **Cytokine Signaling** | **Reactome** | **0.015766182** | **6** | **0.008547009** |
| **Lung Fibrosis** | **WikiPathways** | **3.34E-05** | **4** | **0.063492063** |
| **Macrophage Markers** | **WikiPathways** | **0.025804208** | **3** | **0.020100503** |
| **Toll Like Receptor Signaling** | **WikiPathways** | **6.38E-04** | **3** | **0.25** |
| **IL17 Signaling WP2112** | **WikiPathways** | **6.70E-04** | **3** | **0.050847458** |
| **Acute Myeloid Leukemia** | **WikiPathways** | **0.001014674** | **3** | **0.044117647** |
